# Circumcision for HIV Prevention: Authors' Reply

**DOI:** 10.1371/journal.pmed.0040146

**Published:** 2007-03-27

**Authors:** James G Kahn, Elliot Marseille, Bertran Auvert

The issues regarding risk compensation raised by Kalichman et al. [1] are cogent for refining modeled estimates of the impact of male circumcision (MC). Even more important is to empirically monitor risk compensation during the scale-up of male circumcision.

As Kalichman et al. note, we included in our modeling of MC impact a risk compensation level for men susceptible to HIV above that observed in the Orange Farm trial.

However, we did not incorporate risk compensation among the HIV infected, an adjustment which would have lessened the estimated benefits of male circumcision. These two biases are offsetting. Another conservative bias in our analysis is that we used the per-randomization protective effect of 0.60, rather than the per-clinical protocol protective effect of 0.70. Arguably, effectiveness in practice is better captured by the latter, and this would increase the estimated benefits of male circumcision.

The inclusion of the effects of non-HIV sexually transmitted infections (STIs) as risk co-factors would add a useful dimension to our analysis. The net effect could be to decrease or increase MC impact. As Kalichman et al. note, increased STIs associated with risk compensation in newly circumcised HIV-infected men would likely lessen MC impact. However, in a concentrated epidemic setting where STIs play a greater role in HIV transmission than in South Africa, the STI-reducing effects of MC in HIV-susceptible men could further increase the benefits of MC in preventing HIV.

Regarding the magnitude of risk compensation, we are encouraged by recent data suggesting that MC does not increase risky behavior, and may lead to a transient decrease [2]. However, we, like Kalichman et al. and others, are eager to see the favorable experience in clinical trials carried over to routine and widely operating programs. Thus, the current efforts to plan MC scale-up emphasize the need for an MC procedure that incorporates effective risk reduction counseling. In the context of a medicalized adult male circumcision model, and a clear public health message, risk compensation can be minimized. Thus, a great value of MC scale-up is the opportunity to directly deliver a strong behavioral prevention message. A similar risk reduction message has worked well with antiretroviral therapy in Africa [3].

The ultimate and critical test is monitoring risk behaviors in communities where MC is scaled up. If risk compensation is higher than expected, redoubled risk reduction methods will be imperative.
